# A Newly Observed Mutation of the *ABCA3* Gene Causing Lethal Respiratory Failure of a Full-Term Newborn: A Case Report

**DOI:** 10.3389/fgene.2020.568303

**Published:** 2020-08-31

**Authors:** Martin Jouza, Tomas Jimramovsky, Eva Sloukova, Jakub Pecl, Anna Seehofnerova, Marta Jezova, Milan Urik, Lumir Kunovsky, Katerina Slaba, Petr Stourac, Martina Klincova, Jaroslav A. Hubacek, Petr Jabandziev

**Affiliations:** ^1^Department of Pediatrics, University Hospital Brno, Brno, Czechia; ^2^Faculty of Medicine, Masaryk University, Brno, Czechia; ^3^Department of Pediatric Radiology, University Hospital Brno, Brno, Czechia; ^4^Department of Pathology, University Hospital Brno, Brno, Czechia; ^5^Department of Pediatric Otorhinolaryngology, University Hospital Brno, Brno, Czechia; ^6^Department of Gastroenterology and Internal Medicine, University Hospital Brno, Brno, Czechia; ^7^Department of Surgery, University Hospital Brno, Brno, Czechia; ^8^Department of Pediatric Anesthesiology and Intensive Care Medicine, University Hospital Brno, Brno, Czechia; ^9^Center for Experimental Medicine, Institute for Clinical and Experimental Medicine, Prague, Czechia; ^10^3rd Department of Internal Medicine, 1st Faculty of Medicine, Charles University, Prague, Czechia; ^11^Central European Institute of Technology, Brno, Czechia

**Keywords:** surfactant, *ABCA3*, respiratory distress syndrome, respiratory failure, children

## Abstract

Respiratory distress syndrome caused by a secondary surfactant deficiency is one of the most common diagnoses requiring admission to the Neonatal Intensive Care Unit. We illustrate the case of a term female newborn without prenatal and peripartal risks. There had been significant signs of respiratory distress 4 h after delivery. The condition gradually worsened to the point of needing oscillatory ventilation. The most common infectious and non-infectious causes were excluded. Considering the course of illness, a congenital surfactant deficiency was suspected. There nevertheless was no significant improvement after administration of surfactant. Following a short period of palliative care, the child died at 34 days of age due to respiratory failure. DNA diagnostics revealed compound heterozygosity of *ABCA3* functional mutations leading to the p.Pro147Leu and p.Pro246Leu exchanges. The second identified mutation of *ABCA3* c.737C>T had not to date been described in connection with primary surfactant deficiency.

## Introduction

Respiratory distress syndrome (RDS) is one of the most common diagnoses for which neonates are admitted to the Neonatal Intensive Care Unit (NICU). In the vast majority of these newborns, including both premature and term babies, respiratory distress is caused by surfactant deficiency or its secondary dysfunction. Only a very small percentage of cases is caused by primary surfactant deficiency (Nogee, 2017).

Surfactant is a complex compound of phospholipids and proteins found in the lungs of all mammals. Its main function is to reduce surface tension of the alveoli in order to maintain effective gas exchange in the lungs (Nogee, 2017).

The surfactant consists 90% of phospholipids and 10% of proteins (Gower and Nogee, 2011). The specific surfactant proteins A (SP-A), B (SP-B), C (SP-C), and D (SP-D) are encoded by the *SFTPA*, *SFTPB*, *SFTPC*, and *SFTPD* genes and have considerable functional significance (Hallman, 2013). SP-A and SP-D are structurally similar hydrophilic proteins from the collectin family. SP-B and SP-C, on the contrary, are extremely hydrophobic proteins (Gower and Nogee, 2011). The surfactant is produced by epithelial cells termed type II pneumocytes, and intracellularly it is embedded in inclusion organelles called lamellar bodies (Hallman, 2013). Type II pneumocytes differentiate between weeks 24 and 34 of gestation. Premature newborns with RDS have only about one-tenth the amount of surfactant compared to healthy full-term newborns (Hallman, 2013).

Genetically related surfactant metabolism disorder is a group of diseases spanning a wide range of clinical manifestations, ranging from lethal respiratory failure in neonates to chronic interstitial lung disease (ILD) in children and adults (Griese et al., 2009; Malý et al., 2014).

Mutations of *SFTPB* (OMIM acc. No. 178640), *SFTPC* (OMIM acc. No. 178620), and *ABCA3* (OMIM acc. No. 601615) genes can lead to both qualitative and quantitative surfactant defects (Malý et al., 2014). The most common cause of primary surfactant defect, however, is the loss of function mutations in the ABCA3 gene (Gonçalves et al., 2014).

## Case

We describe here a case of lethal respiratory failure in a term female newborn as a consequence of surfactant metabolism defect. The neonate was born to a 40-year-old mother (gravid 2/para 2). The patient’s mother and father were not consanguineous. Antenatal screenings were all normal. Delivery was induced vaginally at 41 weeks of gestation. The birth weight was 3,150 grams and the Apgar score was of no physiological concern (9-10-10). The first clinical examination of the newborn was without notable pathology. During the first hours of her life, silent grunting, increased salivation, and nasal flaring occurred. Due to the dyspnea and increased oxygen demand, non-invasive respiratory support was first introduced via high-flow nasal cannula. Clinically, there was no significant improvement. Therefore, orotracheal intubation and induction of invasive ventilation were undertaken. All possible and common causes of RDS were consecutively excluded. During bronchoscopy, macroscopic findings of the airways were physiological, the examination of bronchoalveolar lavage revealed the occurrence of lipid-laden macrophages. Diagnosis of surfactant metabolism disorder was then suspected. An exogenous surfactant was therefore administered, after which only a brief improvement in oxygenation occurred.

**FIGURE 1:**
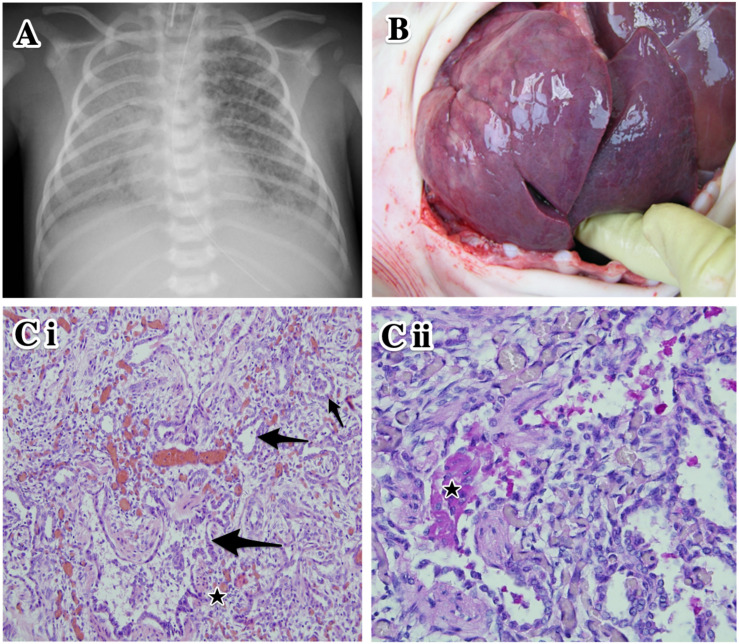
Clinical data of the proband. **(A)** Plain chest X-ray at day 27 of life. High-frequency oscillatory ventilation. Asymmetrical transparency of right lung with signs of developing bronchopulmonary dysplasia. **(B)** Autopsy. Lung parenchyma is airless, pale violet color, tough and rigid to touch. **(Ci)** Histological examination of lung tissue. Marked variable interstitial widening (stars) and diffuse type II pneumocyte hyperplasia (arrows). Hematoxylin–eosin staining, original magnification 100×. **(Cii)** Intra-alveolar glassy eosinophilic homogenous periodic acid-Schiff stain (PAS)-positive material (star). Original magnification 200×.

Due to insufficient ventilation and oxygenation, the ventilation regime was changed on day 24 to high-frequency oscillatory ventilation with fiO2 of 0.8–1.0 (Figure 1A).

On day 34, there was significant deterioration and unsatisfactory saturation despite maximum ventilation. Following discussion with her parents, palliative care was commenced. The girl was extubated and died in the arms of her parents on the 34th day of her life.

At autopsy, the lungs were of pale violet color and, because they were filling the entire thoracic cavity, imprints of the upper ribs were evident. The tissue was firmer and more rigid than usual; under high pressure, a small amount of milky white liquid was oozing from an incision (Figure 1B). The histological finding corresponded to chronic pneumonitis of infancy. In some alveoli, a lumpy, dark, eosinophilic periodic acid-Schiff stain (PAS)-positive material was also found (Figures 1Ci,ii). In the context of the patient’s age and medical history, this histological picture corresponded to congenital deficiency of the surfactant. Remodeling of the lung tissue did not allow effective respiration and the condition was incompatible with life. The autopsy revealed no other developmental defects.

With the informed consent of her parents, the whole blood was sent for next-generation sequencing (NGS) to the Laboratory of Medical Genetics (Hospital Agel, Nový Jičín, Czech Republic). A targeted gene panel covering 484 genes previously associated with the metabolism of surfactant was used for the detection of potential causal mutations. This NGS analysis revealed two heterozygous mutations – c.440C>T (p.Pro147Leu) and c.737C>T (p.Pro246Leu) – in the *ABCA3* gene, potentially associated with a surfactant defect. No additional relevant pathogenic or likely pathogenic mutations were identified by NGS.

**FIGURE 2:**
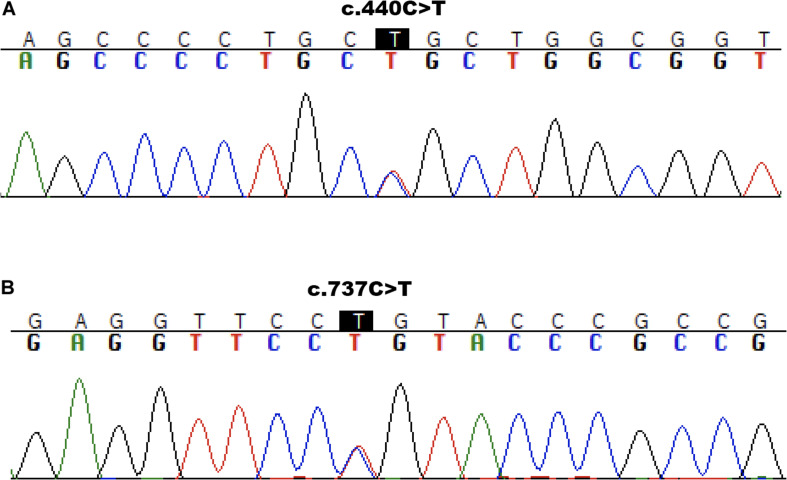
Sanger sequencing results. Molecular analysis presenting ABCA3 variant c.440C>T/p.Pro147Leu to be inherited from mother **(A)** and variant c.737C>T/p.Pro246Leu from father **(B)**.

We further confirmed both variants in the patient’s DNA by targeted Sanger sequencing. In analyzing the parents’ DNA, the c.440C>T mutation was detected in the mother (Figure 2A) and the c.737C>T was found to be inherited from the father (Figure 2B). The c.440C>T variant is described in HGMD (Human Gene Mutation Database) as pathogenic – class 5. As reported in gnomAD (Genome Aggregation Database), the frequency of this mutation in the population is 0.0022% (all heterozygotes). The second identified mutation of *ABCA3* c.737C>T had not to date been described in connection with primary surfactant deficiency and was not listed in the ClinVar and HGMD databases. It has allele frequency in the population of 0.0008% (all heterozygotes) and was predicted *in silico* as deleterious by multiple algorithms and to have a high score for evolutionary conservation. Based on the high scoring for pathogenicity of the variant by five common prediction algorithms and its low frequency in the population, we classify the variant as class 4 – likely pathogenic. Results of predictive algorithms and other features of the identified variants are summarized in Table 1.

**TABLE 1:** Summary of the features of the identified ABCA3 gene variants.

	ABCA3 variants	
	c.440C>T (p.Pro147Leu)	c.737C>T (p.Pro246Leu)
gnomAD frequency	0.0022%	0.0008%
PolyPhen-2	Probably damaging (Score = 1)	Probably damaging (Score = 1)
CADD	Deleterious (Score = 33, high)	Deleterious (Score = 27.8, high)
MutationTaster	Disease-causing (Rank score = 0.81)	Disease-causing (Rank score = 0.81)
SIFT	Damaging (Rank score = 0.913)	Damaging (Rank score = 0.91)
GERP	A high score (RS = 5.42)	A high score (RS = 5.08)
Variant classification	Class 5 (Pathogenic)	Class 4 (Likely pathogenic)

*CADD, The Combined Annotation Dependent Depletion; SIFT, Sorting Intolerant From Tolerant; GERP, Genomic Evolutionary Rate Profiling.*

## Discussion

*ABCA3* mutation appears to be the most common cause of inherited surfactant deficiency (Gonçalves et al., 2014). ABCA3 is a transmembrane phospholipid glycoprotein, a member of the ABC ATP-binding cassette family (AlAnazi et al., 2017).

More than 400 mutations of the *ABCA3* gene have been identified to date, but only a handful have been studied *in vitro* to determine their specific effects on ABCA3 expression, intracellular routing, and/or function (Nogee, 2019). The most common *ABCA3* mutation is p.Glu292Val. It is seen predominantly in patients with a mild form of the disease (Bullard et al., 2005). Biallelic mutation causing congenital surfactant deficiency in a newborn with fatal RDS was first described in 2004 (Shulenin et al., 2004). Wambach et al. suggested that the carrier rate of *ABCA3* mutations could be as high as 1 in 30 individuals of European descent and 1 in 70 individuals of African descent, which projects a disease incidence ranging from 1:4,000 to 1:17,000 individuals. This relatively high incidence but low frequency of reported cases could imply that individuals with milder disease are unrecognized and underdiagnosed (Wambach et al., 2012).

Considering the existence of individuals with milder forms of the disease, this implies that a crucial level of ABCA3 protein is essential for the normal development of lung function. Varieties of *ABCA3* genes could play important roles in the pathogenesis of other lung diseases, including preterm RDS (Polin et al., 2017). Based upon whole-lung lavage of ABCA3-deficient infants prior to lung transplant, Garmany et al. (2006) confirmed decreased amount of surfactant phospholipids and impaired ability to lower surface tension.

ABCA3 mutations are classified into “null” mutations, including non-sense and frameshift mutations predicted to result in truncated or non-functional proteins, as well as “other” mutations, including in-frame insertion/deletion, splice-site, and missense mutations, whose effects on protein function are more difficult to predict. ABCA3 missense mutations might lead to impaired trafficking or dysfunction of ABCA3 protein. Matsumura et al. distinguished type 1 mutations with an abnormal intracellular localization and type 2 mutations with decreased ATP hydrolysis despite correct intracellular localization of ABCA3 protein (Schindlbeck et al., 2018). The compound heterozygous combination of types 1 and 2 mutations is currently discussed as a type 3 mutation and often presents with a more severe phenotype (Beers and Mulugeta, 2017).

Wambach et al. (2014) reported on a study of infants and children with ABCA3 deficiency. All infants in this study had 2 ABCA3 mutations identified. All individuals with null/null had died or undergone lung transplantation by 1 year of age, compared with 62% of the null/other and other/other children. The null/other and other/other subjects presented with respiratory distress at birth, and 23% were still alive at 1 year of age without transplantation. Other than lung transplantation, no specific treatment exists for inherited surfactant deficiency (Malý et al., 2014). Furthermore, mortality and morbidity rates remain high for post-transplant patients (Eldridge et al., 2017).

In our case, confirmation of inherited surfactant deficiency was finally established by sequence analysis of the genomic DNA of the patient and her parents. Two heterozygous mutations – c.440C>T / p.Pro147Leu (class 5 – pathogenic) and c.737C>T / p.Pro246Leu (class 4 – likely pathogenic) – in the *ABCA3* gene, associated with a surfactant defect, were identified in the patient by NGS. By Sanger sequencing, the c.440C>T variant was found to be inherited from the mother and variant c.737C>T from the father. Both amino acid changes are located in the first transmembrane domain of the gene (TMD1) within the first extracellular region (ECD1) of the cell membrane. Missense mutations in the ABCA3 ECD1 region have been shown experimentally to affect the protein trafficking that leads to accumulation of ABCA3 protein in the endoplasmic reticulum (Denman et al., 2018).

## Conclusion

A congenital defect in surfactant metabolism constitutes a very rare disease, but it should be considered especially in term newborns with respiratory failure of unclear etiology. *ABCA3* gene mutations are most commonly associated with a congenital disorder of surfactant metabolism. There is still no causal therapy for this disease today, and most patients afflicted with it die.

Our patient was unfortunate to have inherited two different mutations from her parents, leading to abnormal surfactant function and clinical features of severe RDS. The clinical phenotype and genetic findings support our theory that these mutations were causative rather than neutral variants.

## Data Availability Statement

All datasets generated for this study are included in the article/supplementary material, further inquiries can be directed to the corresponding author.

## Ethics Statement

Written informed consent was obtained from the minor(s)’ legal guardian/next of kin for the publication of any potentially identifiable images or data included in this article.

## Author Contributions

MJo composed the manuscript. TJ, ES, JP, AS, MJe, MU, LK, KS, PS, and MK participated in composing the manuscript. JH interpreted the molecular genetic studies. JH, PS, and PJ proofread the manuscript. All authors read and approved the final manuscript.

## Conflict of Interest

The authors declare that the research was conducted in the absence of any commercial or financial relationships that could be construed as a potential conflict of interest.
